# Anti-Arthritic Effect of the Hydroethanolic Root Extract of *Psydrax subcordata* in Rats

**DOI:** 10.1155/2022/9748382

**Published:** 2022-08-24

**Authors:** Phyllis Annan, Newman Osafo, Paul Poku Sampene Ossei, Wonder Kofi Mensah Abotsi

**Affiliations:** ^1^Department of Pharmacology, Faculty of Pharmacy & Pharmaceutical Sciences, KNUST, Kumasi, Ghana; ^2^Department of Pathology, School of Medicine and Dentistry, KNUST, Kumasi, Ghana

## Abstract

**Background:**

In Ghana, decoctions of various parts of *Psydrax subcordata*, Bridson (Rubiaceae) are employed in the management of inflammatory conditions. However, not much scientific data is available to back such folkloric use of the plant. This study, therefore, seeks to investigate the chronic anti-inflammatory activity of hydroethanolic root extract of *Psydrax subcordata* (PSRE) using the adjuvant-induced arthritis model in rats.

**Methods:**

Freund's adjuvant-induced arthritis model was used to assess the ameliorative effects of PSRE in chronic inflammation. The effect of PSRE on tissue and joint integrity in arthritis was also evaluated by histopathology and microscopy. The effect of PSRE on oxidative markers and serum transforming growth factor (TGF) beta 1 was also determined via chemical assays.

**Results:**

Oral PSRE (30–300 mg/kg) inhibited both ipsilateral and contralateral paw arthritis when given prophylactically and therapeutically in rats. It reduced paw defect on X-ray with histologically-reduced inflammatory cells and synovial hyperplasia. Finally, PSRE significantly reduced TGF-beta 1 levels and raised antioxidants such as reduced glutathione, catalase, and superoxide dismutase levels in arthritic rats.

**Conclusion:**

The findings show that hydroethanolic root extract of *Psydrax subcordata* possesses anti-inflammatory properties in rodents.

## 1. Introduction

Rheumatoid arthritis (RA) is a chronic inflammatory condition that progressively causes bone and cartilage erosion. RA has an impact on health and quality of life [1, 2] which is marked by a chronic course, steady progression, and heightened prevalence of concomitant diseases. All these contribute to the decline in quality of life and eventual disability that accompanies this autoimmune disorder [3]. Some of the established pathological features of RA include joint inflammation of the underlying synovial tissues coupled with tissue hyperplasia [4].

There is a complex aetiopathogenesis of RA which is continuously being investigated [5]. Currently, nonsteroidal anti-inflammatory drugs (NSAIDs), disease-modifying antirheumatic drugs (DMARDs), biological agents, and corticosteroids are the principal agents employed in the management of RA. These agents are used in an attempt to alleviate the symptoms of the disease as well as modulate the disease progression [6]. Although these classes of agents do offer significant therapeutic benefits, the search continues for alternative agents that would also contribute to the effective management of RA.

Plants are widely used in the management of inflammatory conditions among Africans [7]. They do offer a cogent basis for the investigation into potential alternative therapies for inflammatory conditions. One such plant used in the management of chronic inflammatory conditions among the natives in Ghana is *Psydrax subcordata*.

*Psydrax subcordata DC* is a tree that grows mainly in central and western Africa and reaches a height of more than 5–15 m. With a palm-like habit, the branches are often hollow and swollen with access pores and leaves that are broadly at the apex. In Ghana, *Psydrax subcordata* is called ‘ntatiadupon' in Asante-Twi [8]. It is found in tropical rain forests. Its roots, leaves, and stem barks are used for medicinal purposes. Various parts of the plant have been identified to contain compounds such as iridoids as well as shanziside methyl ester isolated from the stem bark and fruit extracts [9].

The plant is known to have wide-ranging therapeutic benefits including, anticonvulsant activity [10], antimicrobial effects [8, 11], and acute anti-inflammatory activity [8]. However, there is currently no study validating its folkloric use in the management of chronic inflammatory conditions. The current study, therefore, sets out to investigate the pharmacological potential of the hydroethanolic root extract of *Psydrax subcordata* in the management of arthritis using animal models.

## 2. Materials and Methods

### 2.1. Plant Collection and Extraction

The roots of the plant *Psydrax subcordata* were collected from the Ashanti-Mampong forest in August 2017. The sample was authenticated at the Department of Herbal Medicine, KNUST and a voucher specimen was kept at the Department's herbarium (KNUST/HM/2016/R002). The roots were air-dried indoors for two weeks. About 914.5 g of the dried root was ground into powder using a heavy-duty blender. Extraction was done with 70% ^v^/_v_ ethanol (5 L) by maceration for 24 h. The ethanol ﬁltrate was concentrated using a rotary evaporator (R-210, BUCHI, Switzerland) and further dried in an oven (Gallenkamp OMT, SANYO, Japan) to yield a 43.4 g solid mass (4.745% (^w^/_w_) yield). The dried extract was reconstituted with normal saline and referred to as PSRE.

### 2.2. Experimental Animals

Sprague Dawley rats (300–350 g) were purchased from Noguchi Memorial Institute for Medical Research (NMIMR), University of Ghana, Accra, Ghana, and kept in the animal facility of the Department of Pharmacology, College of Health Sciences, KNUST, Kumasi, Ghana. All animals were humanely handled in the studies conducted in accordance with internationally accepted principles for laboratory animal use and care (NIH publication #85–23, revised in 1985). Additionally, all animal experiments were approved by the Departmental Research Ethics Committee. Animals were acclimatized for a week before being assigned randomly to their respective groups. Rats or mice (5/cage) were housed in a polypropylene cage and subjected to a 12-h light/dark cycle. The animals had access to commercially acquired chow (Agricare Ltd., Kumasi, Ghana) and water freely. Each animal was used once.

### 2.3. Chemicals and Reagents

Parafﬁn oil was purchased from KAMA Pharmaceutical Industries (Accra, Ghana). Trichloroacetic acid (TCA), thiobarbituric acid (TBA), potassium dichromate, sodium bicarbonate, sodium dihydrogen orthophosphate monohydrate, chloroform, and disodium hydrogen phosphate were purchased from BDH Chemicals (Poole, England). Complete Protease Inhibitor Cocktail Tablet was purchased from Santa Cruz Biotechnology (Dallas, TX, USA). Diclofenac, morphine, naloxone, and xylene were purchased from Sigma Aldrich (St. Louis Missouri, USA).

### 2.4. Microorganism

Heat-killed *Mycobacterium tuberculosis* [strains C, DT and PN (mixed)] was a donation from the Ministry of Agriculture, Fisheries and Food, UK.

### 2.5. Adjuvant-Induced Arthritis in Rats

Adjuvant-induced arthritis in rats was performed as described by Obiri et al. [12]. Briefly, the right hind paw of rats was injected intraplantar with 100 *µ*l of Complete Freund's Adjuvant (CFA). The CFA was prepared as a suspension of 5 mg ml^−1^ of heat-killed *mycobacterium tuberculosis* [strains C, DT, and PN (mixed)] in paraffin oil. The arthritic control group received only intraplantar injection of CFA, while the non-arthritic control/IFA group received only intraplantar injection of 100 *µ*l Incomplete Freund's Adjuvant (IFA) (sterile paraffin oil).

#### 2.5.1. Edema Assessment

The foot volume of the rats was measured by water displacement plethysmography [13] for both the ipsilateral (injected hind paw) and the contralateral paw (non-injected hind paw) before intraplantar injection of CFA or IFA (day 0) and every day up to the 28^th^ day. To quantify the edema component of the induced inflammation, the difference in foot volume between day 0 and the various time points were recorded.

To determine the mean percentage change in paw volume for each treatment, the following equation was used:

(1) $$\begin{array}{c} \%\text{ Change in paw volume}=\frac{\left[\text{PVT}-\text{PV0}\right]}{\text{PV0}}\times 100\%, \end{array}$$

where PV_0_ and PV_T_ are the paw volumes before induction of arthritis, and at the time, T, of the induction of arthritis, respectively.

Total edema induced was determined as the area under the curve (AUC).

To determine the percentage inhibition of total edema for each treatment, the following equation was used:

(2) $$\begin{array}{c} \text{Percentage inhibition of oedema}=\frac{\left[\text{AUC}\left(\text{control}\right)-\text{AUC}\left(\text{treatment}\right)\right]}{\text{ AUC}\left(\text{control}\right)}\times 100\text{\%.} \end{array}$$

Drug effects were determined by comparing the maximal and total edema responses attained during 28 days in drug-treated groups with the corresponding values attained in saline-treated inﬂamed control groups.

*(1) Prophylactic Protocol*. Sprague Dawley rats were put randomly into six groups (*n* = 6) and treated as follows:

  Group I: arthritic control/CFA (intraplantar injection of 100 *µ*l CFA)
  Group ΙI: non-arthritic control/IFA (intraplantar injection of 100 µl of IFA)
  Groups III-V: treated with PSRE (30–300 mg kg^−1^*p.o.*) 1 h before intraplantar injection of CFA and then followed with daily administration up to day 28.
  Groups VI: treated with dexamethasone (0.3 mg kg^−1^ i.p.) 30 min before intraplantar injection of CFA and then followed with daily administration up to day 28.

*(2) Therapeutic Protocol*. Arthritis was induced on day 0 while treatment commenced after the polyarthritis phase has set in from day 14 up to day 28. The animals were grouped (*n* = 6) randomly and treated as follows:

  Group I: arthritic control/CFA (intraplantar injection of 100 *µ*l CFA)
  Group ΙI: non-arthritic control/IFA (intraplantar injection of 100 µl of IFA)
  Groups III-V: treated with PSRE (30–300 mg kg^−1^*p.o.*) daily from day 14 after intraplantar injection of CFA up to day 28.
  Groups VI: treated with dexamethasone (0.3 mg kg^−1^ i.p.) daily from day 14 after intraplantar injection of CFA up to day 28.

Drug effects were determined by comparing the maximal and total edema responses attained during 28 days in drug-treated groups with the corresponding values attained in saline-treated inﬂamed control groups.

#### 2.5.2. Radiological Analysis and Assay of Transforming Growth Factor Beta 1 Levels

On day 29, rats were anesthetized by intraperitoneal injection of 50 mg kg^−1^ pentobarbitone sodium and radiographs taken of the hind limbs. The X-ray apparatus was operated at a 30-kV peak and 10-s exposure with a 45-cm tube-to-film distance for lateral projections [14]. X-ray images were blindly scored on a 0–4 scale by looking at the extent of osteoporosis, joint spaces, osteophytes, and joint structure [15]:

(0) Uninjected control group with no degenerative joint changes
(1) Slight soft tissue volume, joint space, subchondral erosion, periostitis, osteolysis, subluxation, and degenerative joint changes
(2) Low to moderate soft tissue volume, joint space, subchondral erosion, periostitis, osteolysis, subluxation, and degenerative joint changes
(3) Pronounced soft tissue volume, joint space, subchondral erosion, periostitis, osteolysis, subluxation, and degenerative joint changes
(4) Excess soft tissue volume, joint space, subchondral erosion, periostitis, osteolysis, subluxation, and degenerative joint changes

Blood samples were taken and sera were obtained and stored at −20°C. Later, the frozen sera were thawed to room temperature, and TGF-beta 1 was assayed according to the manufacturer's protocol using ab119558 TGF-beta 1 Rat ELISA Kit (Abcam, UK).

### 2.6. Hematological Analysis

The peripheral blood proﬁle changes were analyzed on day 29 of the study. Rats were euthanized and blood samples were collected from the jugular vein. A full blood count was done using an auto hematology analyzer (Mindray BC-2800, Mindray Company, Shenzhen, China). The erythrocyte sedimentation rate (ESR) was also determined using Westergren pipettes (Henso Medical Ltd. Hangzhou, China).

### 2.7. Histopathological Analysis

The bones of the hind paws were excised on day 29 for histological analysis. Excised bones of the ipsilateral limbs were ﬁxed in sterile Bouin's ﬂuid (1% picric acid, 9.5% formaldehyde, 5% acetic acid), decalciﬁed, sectioned, and stained with hematoxylin and eosin. Stained tissues were examined under a microscope (Model BX51, Olympus America Inc., Center Valley, PA) for histopathological changes in joints.

### 2.8. Thermal Hyperalgesia in Arthritic Rat Paws

The anti-hyperalgesic effect of PSRE was investigated using the Hargreaves test [16, 17]. Hind paw sensitivity to a noxious thermal stimulus was measured with the radiant heat source method using the Analgesia Meter (Model 336, IITC Life Science Inc., Woodland Hills, CA, USA). Paw withdrawal latencies (PWLs) were measured at pre-CFA and then daily for 28 days in both protocols. Experiments began by habituating the rats to the Hargreaves chamber for 15 min prior to testing and paw withdrawal latency (PWL) was measured three times on the ipsilateral paw of rats. The thermal stimulus was maintained at an intensity of 80%, increasing temperature to a maximum of 55°C in 40 s. The thermal stimulus was cut off if the rat failed to withdraw its hind paw to prevent tissue injury. A mean of the three recordings was then calculated to give a single measure for each time point. The stimulus intensity and cut-off time were selected, so changes in withdrawal latency were detected while no tissue damage was induced.

### 2.9. Antioxidant Activity

Antioxidant enzyme and oxidative stress assays were conducted on sera obtained from rats in the arthritis test.

*(1) Catalase (CAT) Assay*. The test principle is based on the propensity of CAT to hydrolyze H_2_O_2_, thereby inhibiting the reduction of dichromate in acetic acid to chromate acetate by H_2_O_2_ [18]. Successive additions of 0.4 ml H_2_O_2_ (1.18 M) and 1 ml phosphate buffer (0.01 M; pH 7.0) were made to 0.1 ml of serum and incubated for 5 min (25^o^C). An amount of 2 ml dichromate-acetic acid mixture (containing 3 parts glacial acetic acid and 1 part 5% potassium dichromate) was added to terminate the reaction. A pipetted volume of 150 µl of the mixture was taken into a 96-well plate. Absorbance was read with a spectrophotometer (Synergy H1 Multi-mode Reader, BioTek Technologies, Winooski, VT, USA) at 620 nm. Specific CAT activity was expressed as units per mg protein based on the molar extinction coefficient of H_2_O_2_, 39.4 M^−1^ cm^−1^ at 620 nm.

*(2) Superoxide Dismutase (SOD) Assay*. This test works on the principle that adrenaline autoxidation to adrenochrome is prevented in the presence of SOD [19]. A volume of 500 *μ*l rat serum was centrifuged in 150 *μ*l of ice-cold chloroform and 750 *μ*l ethanol (96% v/v) at 2000 ×g for 20 min. Successive additions of 1 ml carbonate buffer (0.1 M; pH 10.2) and 0.5 ml EDTA (0.6 mM) were made to 500 *μ*l of supernatant aliquot. An amount of 0.05 ml of 1.3 mM adrenaline solution was then added to initiate adrenochrome formation. A blank solution containing all reagents (except serum) was processed similarly. A pipetted volume of 150 *µ*l was then dispensed into a 96-well plate. Absorbance was read using a spectrophotometer (Synergy H1 Multi-mode Reader, BioTek Technologies, Winooski, VT, USA) at 480 nm.

*(3)*. *Reduced Glutathione (GSH) Assay.* The concentration of GSH in rat serum was measured by the procedure described by Ellman [20]. To 100 *µ*l of the serum was added 2.4 ml 0.02 M EDTA, and then cooled at 4 °C for 10 min. An amount of 2 ml H_2_O and 0.5 ml of 50% ^w^/_v_ TCA were added to the mixture and centrifuged at 3000 × g for 5 min. About 50 *μ*l of 10 mM DTNB solution and 2 ml of Tris buffer (0.4 M; pH 8.9) were then thoroughly mixed with 1 ml of the supernatant and the reaction was incubated for 5 min (25 °C). A reaction mixture was repeated also for the blank. A 96-well plate was filled with 150 *µ*l of mixture and absorbance was spectrophotometrically (Synergy H1 Multi-mode Reader, BioTek Technologies, Winooski, VT, USA) read at 412 nm. GSH concentration was expressed in µmol per mg protein and determined using the equation  *y* = 0.0004 *x* + 0.0026.

### 2.10. Statistical Analysis

All data are presented as the means ± SEM. Data were analyzed using GraphPad Prism for Windows Version 7.00 (GraphPad Software, San Diego, CA, USA). Data for adjuvant-induced arthritis for the 28 days are presented as the time-course curves. The time course curves were subjected to a two-way (*treatment* × *time*) repeated measures analysis of variance with Tukey's *post hoc* test. Differences in AUCs were analyzed by ANOVA followed by Tukey's post hoc test.

## 3. Results

### 3.1. Edema Assessment

**Figure 1:**
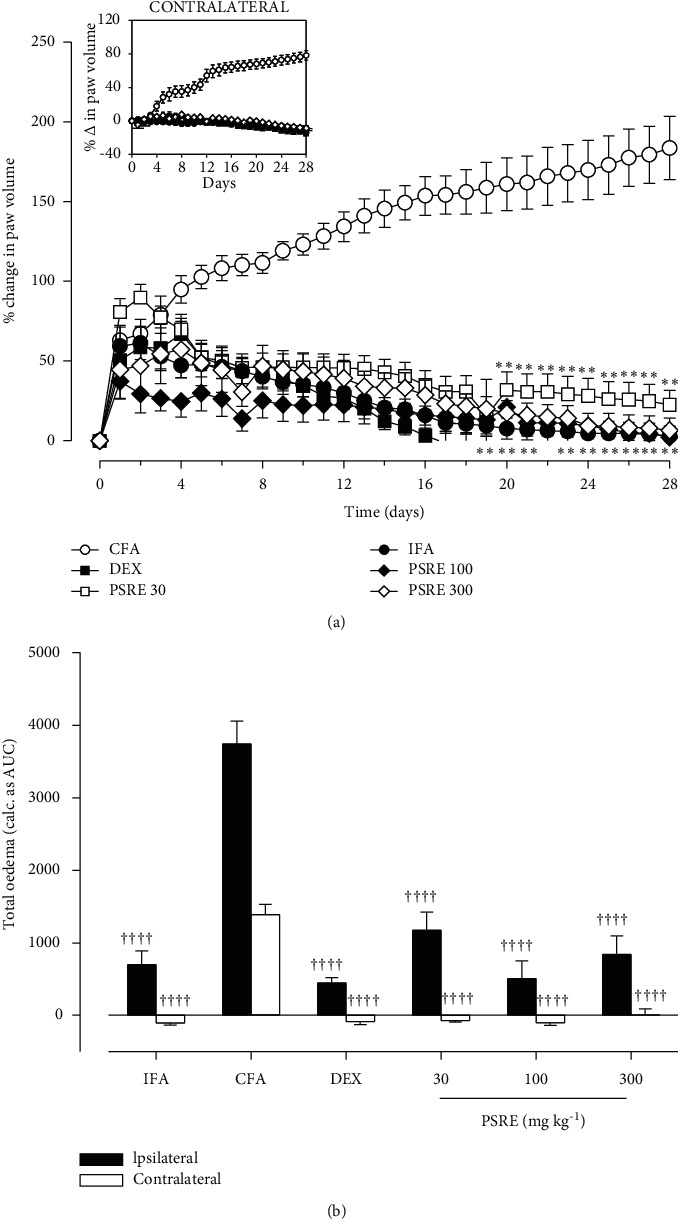
Effect of prophylactic administration of PSRE and dexamethasone on time-course curves (a) and total paw edema (b) in adjuvant-induced arthritis in rats. Data is presented as Mean ± S.E.M. (*n* = 5–6). ^*∗∗*^*P* < 0.01 compared to CFA group (Two-way ANOVA followed by Tukey's multiple comparison test). ^††††^*P* < 0.0001 compared to CFA group (Tukey's multiple comparison test).

**Figure 2:**
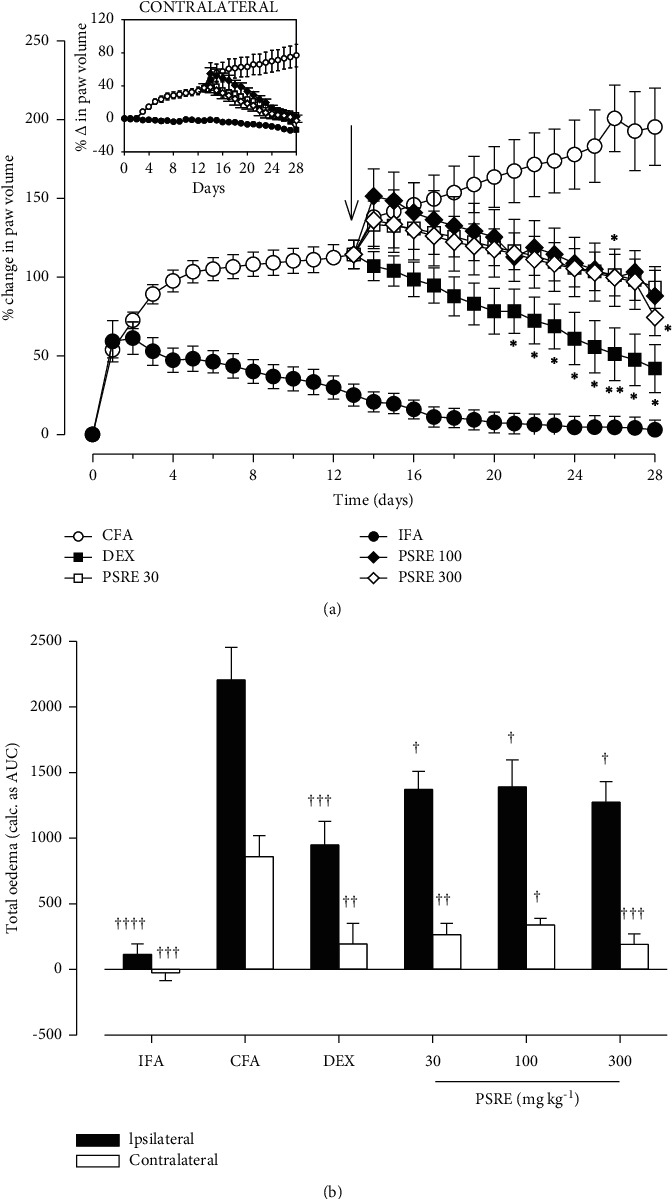
Effect of therapeutic administration of PSRE and dexamethasone on time-course curves (a) and total paw edema (b) in adjuvant-induced arthritis in rats. Data is presented as Mean ± S.E.M. (*n* = 5–6). Drugs were administered from day 14 to day 28. ^*∗*^*P* < 0.05, ^*∗∗*^*P* < 0.01 compared to the CFA group (Two-way ANOVA followed by Tukey's multiple comparison test). ^†^*P* < 0.05, ^††^*P* < 0.01, ^†††^*P* < 0.001, ^††††^*P* < 0.0001 compared to CFA group (Tukey's multiple comparison test).

From the results obtained, rats in the IFA group expectedly showed no signiﬁcant change in paw volume throughout the study (Figures 1 and 2). Injection of Freund's adjuvant into rats' hind paws resulted in sudden and continuous swelling of the injected (ipsilateral) hind paw. The edema progressed during the acute inflammation phase on days 4–7, followed by chronic inflammation and systemic spread (polyarthritis) to contralateral paws from days 10–14 until the 28th day (Figures 1(a) and 2(a)). The total paw swellings induced over the 28 days were measured as the area under the time course curve, AUC for ipsilateral and contralateral paws respectively (Figures 1(b) and 2(b)).

In the prophylactic protocol, where drug treatment was started before the induction of arthritis, all doses of PSRE and dexamethasone (0.3 mg kg^−1^) used significantly (*F*_5, 27_ = 14.33, *P* < 0.0001) affected the time–course curve of paw edema. Ipsilateral paw volumes decreased from day 8 to day 28 when compared to the CFA control rats (Figure 1(a)). All doses of PSRE and dexamethasone used also significantly (*F*_5, 27_ = 69.74, *P* < 0.0001) prevented the spread of arthritis to the contralateral paw (Figures 1(a) and 1(b)).

In a separate experiment (therapeutic protocol), drug treatment began on day 14. Treatment of rats with PSRE (30–300 mg kg^−1^) and dexamethasone (0.3 mg kg^−1^) significantly (*F*_5, 24_ = 14.92, *P* < 0.0001) reduced ipsilateral paw swelling from day 16 to day 28 compared with the arthritic (CFA) control rats (Figures 2(a), 2(b)). PSRE (30–300 mg kg^−1^) also significantly (*F*_5, 28_ = 8.223, *P* < 0.0001) reduced edema in the contralateral paw by day 28 (Figure 2(b)). Likewise, dexamethasone (0.3 mg kg^−1^) significantly (*P* < 0.001) attenuated systemic arthritis spread depicted as a reduction in contralateral paw swelling (Figures 2(a) and 2(b)).

With similarity in the observations made in both prophylactic and therapeutic protocols of adjuvant-induced arthritis studied, the subsequent assessments, henceforth, report only on the therapeutic protocol.

### 3.2. Radiological Analysis and Assay of Transforming Growth Factor Beta 1 (TGF-Beta 1)

**Figure 3:**
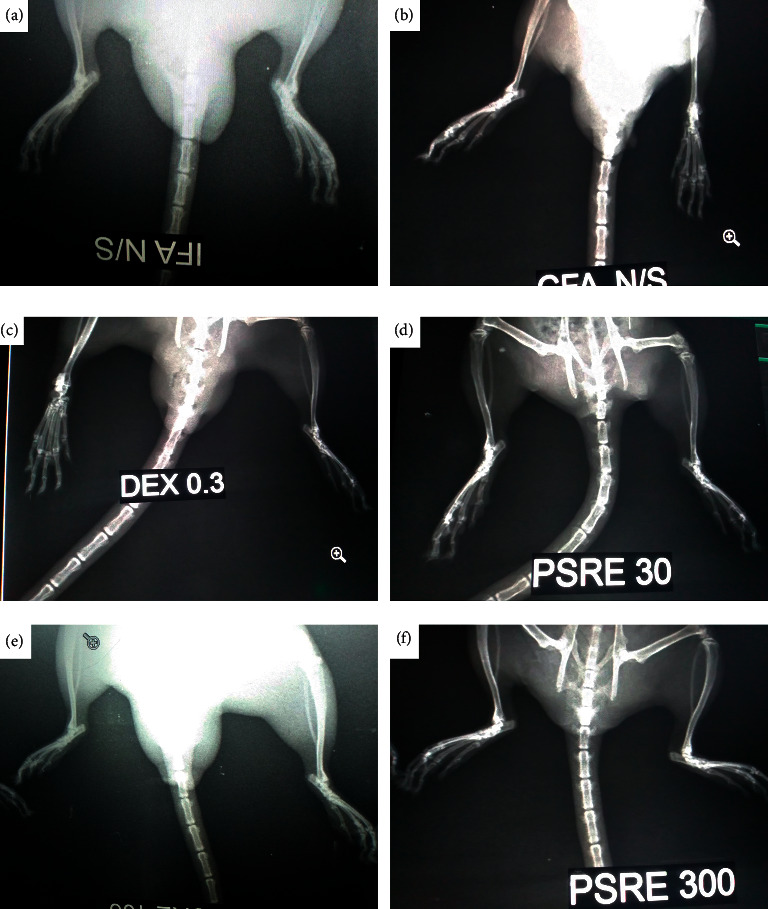
Radiographs of rats pretreated with IFA (a), CFA (b), dexamethasone (c), 30 mg kg^−1^ of PSRE (d), 100 mg kg^−1^ of PSRE (e) and 300 mg kg^−1^ of PSRE (f) in adjuvant-induced arthritis.

To study the extent of joint damage in arthritis, radiographs were taken. IFA control rats in the study presented with no osteophytes in the bone metaphysis. Generally, there were signiﬁcantly reduced signs of bone deformation when rats were treated with PSRE at doses of 100 and 300 mg kg^−1^ (Figures 3(e) and 3(f)).

**Figure 4:**
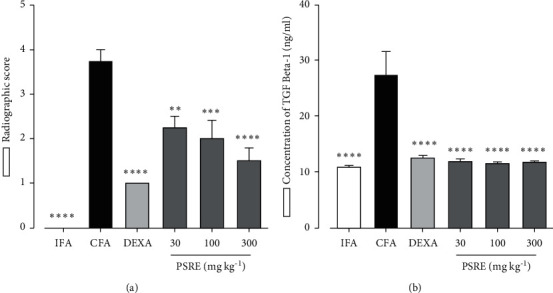
Effect of PSRE and dexamethasone on radiographic scores (a) and TGF-beta1 levels (b) in rats in the therapeutic protocol of adjuvant-induced arthritis. Values are the mean ± SEM. (*n* = 5–6). ^*∗∗*^*P* < 0.01, ^*∗∗∗*^*P* < 0.001, ^*∗∗∗∗*^*P* < 0.0001 compared to CFA group (One-way ANOVA analysis followed by Tukey's multiple comparison test).

Figure 4(a) shows the radiographic scores obtained from rats in the therapeutic adjuvant-induced arthritis study. Based on the scores, CFA control rats exhibited the most joint destruction following inflammation when compared to the IFA group (*P* < 0.0001). Arthritic rats given PSRE and dexamethasone (0.3 mg kg^−1^) showed significantly less damage to the joints (Figure 4(a)).

The concentrations of TGF-beta 1 were significantly reduced (*F*_5, 18_ = 12.92, *P* < 0.0001) in the IFA, dexamethasone and PSRE-treated groups when compared to the CFA control rats (Figure 4(b)).

### 3.3. Hematological Analysis

There was a significant decrease in ESR for non-arthritic rats (IFA group) whiles the arthritic rats (CFA group) had an increase in ESR (*F*_5, 12_ = 12.73, *P*=0.0001). PSRE significantly (*P* < 0.0001) decreased ESR, however, it did not significantly alter the HGB, RBC, and HCT levels (Table 1). There was again a significant increase in platelets (*P*=0.0368) and a decrease in lymphocytes (*P*=0.0005) in dexamethasone-treated rats compared to the arthritis controls (Table 1).

When serum chemistry was done, the extract showed no significant changes in all assayed parameters(Table 2).

### 3.4. Histopathological Analysis

**Figure 5:**
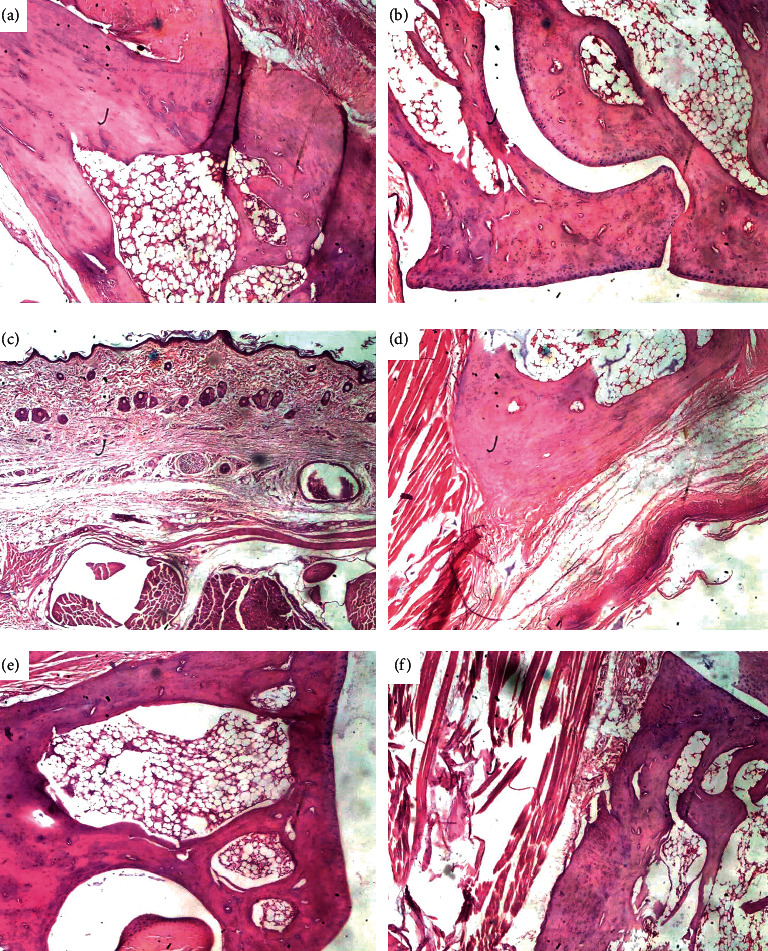
Photomicrographs from the joints of rats pretreated with IFA (a), CFA (b), dexamethasone (c), 30 mg kg^−1^ of PSRE (d), 100 mg kg^−1^ of PSRE (e) and 300 mg kg^−1^ of PSRE (f) in adjuvant-induced arthritis.

IFA-treated control rats had an intact bone structure with no signs of inflammatory cell infiltration. In the CFA-treated control rats, there was mononuclear cell inﬁltration and vascularity in synovial tissues with characteristic redness and pannus invasion. Also, cartilage destruction and severe synovial hyperplasia of the subchondral bone were visible (Figure 5). Rats therapeutically treated with dexamethasone showed signs of synovial hyperplasia, severe thin-walled epithelium, preserve skin appendages, moderate fibromuscular connective tissues, and moderate to severe bone erosion. When arthritic rats were therapeutically treated with 30 mg kg^−1^ PSRE, there was a markedly thinned layered *epidermis*, hair follicles atrophic, moderate keratosis, marked hyalinized fibromuscular connective tissue, and dilated blood vessels that were congested. There was also evidence of severe bone erosion, and with moderate osteocyte formation and granulation tissue formation. When 100 mg kg^−1^ of PSRE was administered, rats showed moderate bone erosion and synovial hyperplasia. There was moderate to severe cartilage destruction as well. 300 mg kg^−1^ administration resulted in signs of bone healing and dense bone with mild erosion with no evidence of synovial hyperplasia (Figure 5).

### 3.5. Thermal Hyperalgesia in Arthritic Rat Paws

**Figure 6:**
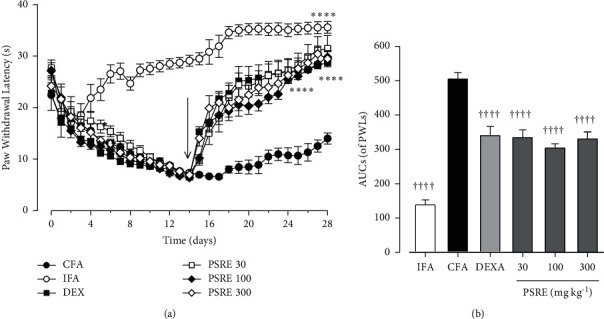
Effect of PSRE (30–300 mg kg^−1^) and dexamethasone (0.3 mg kg^−1^) on time-course curves of paw withdrawal latencies (a) and AUCs (b) in the therapeutic protocol of adjuvant-induced arthritis in rats. Values represent mean ± S.E.M (*n* = 5–6). ^∗∗∗∗^*P* < 0.0001 compared to the CFA group (Two-way ANOVA followed by Tukey's multiple comparison test). ^††††^*P* < 0.0001 compared to CFA group (One-way ANOVA analysis followed by Tukey's multiple comparison test).

The paw withdrawal latencies (PWLs) of IFA control rats increased from the baseline of 27.22 ± 2.11 s to 33.55 ± 6.34 s on day 28. CFA-treated control rats, however, had a reduction in the day zero (baseline) reading of 28.0 ± 7.2 s to 6.5 ± 4.23 s on day 14 and a gradual rise to 13.9 ± 9.41s on day 28. Treatment of rats with PSRE (30 and 100 mg kg^−1^) and dexamethasone (0.3 mg kg^−1^) from day 14 significantly increased PWLs compared to CFA controls (Figure 6(a) and 6(b)). PWL for PSRE 300 mg kg^−1^ group rose sharply after treatment began on day 14 from 14.02 ± 7.73 s to 29.4 ± 4.75 s on day 28 (Figure 6(a)).

### 3.6. Antioxidant Activity

#### 3.6.1. Catalase (CAT) Assay

**Figure 7:**
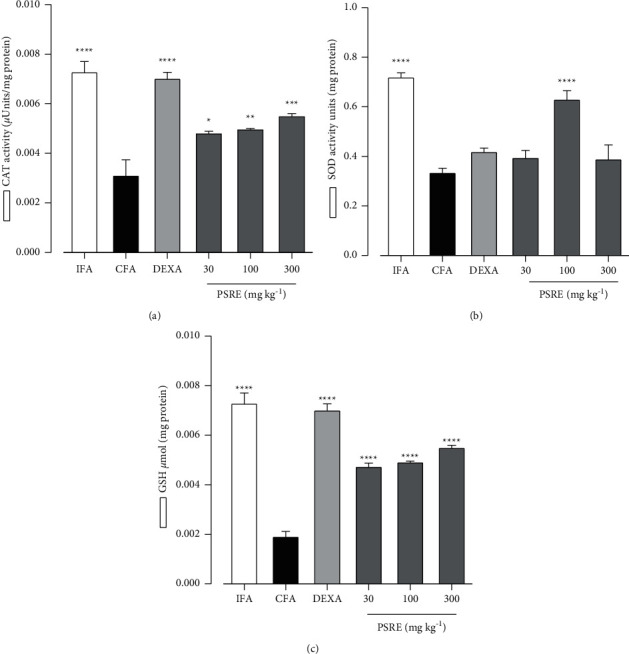
The effect of PSRE and dexamethasone on serum CAT (a), SOD (b) and GSH (c) activities of rats in the therapeutic adjuvant arthritis study. Values are the mean ± SEM (*n* = 5–6). Significantly different values: ^*∗*^*P* < 0.05, ^*∗∗*^*P* < 0.01, ^*∗∗∗*^*P* < 0.001, ^*∗∗∗∗*^*P* < 0.0001 compared to CFA group (One-way ANOVA analysis followed by Tukey's multiple comparison test).

In the therapeutic studies, catalase activity seen in IFA control rats was significantly (*P* < 0.0001) higher than that of CFA-treated control rats (Figure 7(a)). Similarly, PSRE (30, 100, and 300 mg kg^−1^) and dexamethasone 0.3 mg kg^−1^ treated rats also showed significantly (at least *P* < 0.05) increased serum CAT activity (Figure 7(a)).

#### 3.6.2. Superoxide Dismutase (SOD) Assay

The CFA-challenge significantly reduced (*P* < 0.001) SOD activity in rats compared to that seen in IFA-treated control rats (Figure 7(b)). There was no significant change in SOD activity upon administration of dexamethasone. At 100 mg kg^−1^ dose of PSRE, there was a significant (*F*_2, 24_ = 23.21, *P* < 0.0001) increase in SOD enzyme activity (Figure 7(b)).

#### 3.6.3. Reduced Glutathione (GSH) Assay

The levels of GSH in the arthritic (CFA) group were significantly decreased (*P* < 0.0001) compared to non-arthritic control (IFA) rats (Figure 7(c)). Treatment with PSRE (30, 100, and 300 mg kg^−1^) and dexamethasone 0.3 mg kg^−1^ significantly (*P* < 0.001) increased reduced GSH levels (Figure 7(c)).

## 4. Discussion

This current study has demonstrated that the hydroethanolic root extract of *Psydrax subcordata* (DC.) Bridson has anti-inflammatory properties in Freund's adjuvant model of chronic inflammation in rats.

A widely used model for preclinical chronic inflammation investigation is Freund's adjuvant-induced inflammation model [21]. The role of autoantigens that cross-react with the *Mycobacterium* injected has been widely documented to account for the disease pathogenesis [22]. The pathological parameters of the model which include angiogenesis, chondrocyte degradation, bone erosion, and infiltration of inflammatory cells are similar to rheumatoid arthritis (RA) in humans [12, 23]. This makes the model reliable and widely employed in the investigation of potential anti-RA drugs. This study investigated the potential benefits of PSRE in the management of rheumatoid arthritis.

Swelling of the rat paws after subplantar CFA-challenge is a sensitive and rapid indicator for assessing the extent of inflammation in arthritis and the potential of antiarthritic drugs to reduce inflammation [24, 25]. Further, the radiographical analysis offers true remission of the disease and an accurate assessment of the status of the disease [12]. In Adjuvant arthritis, There is increased bone resorption with the reduction in the rate of new bone formation resulting in significant bone loss [26]. The disease progression is multifaceted and effectively has an induction phase, early synovitis, and late synovitis. A potential RA treatment has the potential of blocking at least two of these phases [12]. In our current study, the PSRE was able to curtail the inflammation and synovitis and was able to reduce the spread of the edema to the uninjected paw. Also, administration of PSRE to rats was able to reduce the severity of joint deformation in both preventive and therapeutical protocols hence indicated its benefits in RA.

RA is marked by a significant elevation of WBC which is an indication of inflammation associated with the infection [27]. Also, accompanying RA is an elevated ESR in 95% of clinical cases albeit not specific for RA [28]. The induced inflammation results in elevated proportions of fibrinogen in the blood resulting in the clumping of the blood cells hence elevating the ESR. With PSRE administration, there was an effective reduction in ESR and observed reduction in WBC, albeit not statistically significant, indicative of its benefits in inflammation management.

TGF-beta 1 is abundantly expressed in RA. The elevated levels of TGF-beta 1 in the rat synovium in the study indicate the important role of the cytokine in the disease progression [29]. Although the immune-suppressive and anti-inflammatory properties of TGF-beta 1 are known, its potential pro-inflammatory importance in RA is possible [30]. Earlier studies by Allen et al. [31] and Olsen et al. [32] showed that injection of TGF-beta 1 into the synovium of arthritis rats induced an inflammatory outcome marked by neutrophil accumulation and worsening of the arthritic response. Also, anti-TGF-beta antibody blocked the accumulation of inflammatory cells and tissue pathology when assessed in an experimental model of chronic erosive polyarthritis in a study by Wahl et. al [30]. It was therefore not surprising to see elevated TGF-beta 1 levels in arthritic rats from our study. PSRE when administered therapeutically reduced the expression of TGF-beta 1 hence contributing to its potential pathway(s) of chronic inflammation inhibition.

In RA assessment, histopathology provides a good morphological distinctiveness of the disease progress. Histopathological assessment of the disease gives a good indication of the bone destruction that is a hallmark of the condition [33]. RA traditionally is marked by profound erosion, significant mononuclear cell infiltration, and vascularity of synovial tissues. PSRE reducing the extent of bone erosion and infiltration of inflammation cells in our study is consistent with earlier studies that established the appropriateness of potential anti-RA drugs to reduce inflamed joints and synovitis, and generally offer joint protection [12, 14, 34].

Thermal hyperalgesia associated with rheumatoid arthritis is increased by the role of protein tyrosine phosphatase 1B. This phosphatase present in the dorsal horn of the spinal cord stimulates Src kinase and promotes NMDA subtype receptor phosphorylation resulting in the excitation of glutamatergic synapse and accounting for the hyperalgesia [35]. Iridoids such as subcordatanols I-IV, 1-*o*-methylcrescentin I, subcordatalactones A and B have been successfully isolated from *Psydrax subcordata* and have shown PTP 1B inhibition from studies by Zhou et al. [36]. It is therefore not surprising to observe an inhibition of inflammatory pain in rats given PSRE. The pain modulation seen in the arthritic rats after PSRE administration may be associated with the PTP 1B nociceptive role.

Rheumatoid arthritis is always associated with oxidative damage [37–39]. From our study, induction of arthritis resulted in reduced expression of catalase and superoxide dismutase as well as the reduction in levels of reduced glutathione. There was however an increase in catalase, superoxide dismutase expression, and increased reduced glutathione levels in arthritis rats after PSRE administration. PSRE is known to be rich in antioxidant principles such as tannins, flavonoids and triterpenoids realized from earlier studies conducted on the plant. These account for the extract's potential to reduce the extent of oxidative damage and contribute to its anti-inflammatory activity.

## 5. Conclusion

We glean from our current study the suppressive effect of the hydroethanolic root extract of *Psydrax subcordata* on inflammation and disease progression in adjuvant-induced arthritis in rats. This gives scientific evidence to the traditional use of the plant in the management of inflammatory conditions.

## Figures and Tables

**Table 1:** Hematological analysis of arthritic rats in the therapeutic study.

Parameters	IFA	CFA	PSRE 30	PSRE 100	PSRE 300	Dex	*F*	*P* Value
ESR (mL/h)	1.00 ± 0.00^c^	3.00 ± 0.00	1.17 ± 0.00^c^	1.10 ± 0.00^c^	1.07 ± 0.17^c^	1.46 ± 0.33^c^	*F*_5, 12_ = 12.73	0.0001
WBC (×10^9^/L)	5.93 ± 0.57	12.53 ± 0.21	9.00 ± 1.62	10.33 ± 2.39	12.53 ± 0.24	9.90 ± 3.30	*F*_5, 12_ = 1.422	0.0285
RBC (×10^12^/L)	7.65 ± 0.22	7.31 ± 0.12	6.39 ± 0.71	7.34 ± 0.38	7.00 ± 0.26	6.39 ± 0.71	*F*_5, 12_ = 1.168	0.5556
HB (g/dL)	14.17 ± 0.43	13.33 ± 0.19	13.37 ± 0.62	13.70 ± 0.23	12.73 ± 0.38	12.00 ± 0.80	*F*_5, 12_ = 2.403	0.0991
HCT (%)	51.37 ± 2.24	49.03 ± 0.73	48.67 ± 3.21	49.93 ± 0.74	45.83 ± 0.96	42.57 ± 3.02	*F*_5, 12_ = 2.299	0.1103
MCV (fL)	67.07 ± 1.02	67.17 ± 2.15	65.40 ± 0.66	68.30 ± 2.65	65.60 ± 1.85	67.40 ± 3.90	*F*_5, 12_ = 0.235	0.9396
MCH (pg)	18.50 ± 0.12	18.27 ± 0.38	18.00 ± 0.38	18.73 ± 0.67	18.20 ± 0.57	18.27 ± 0.38	*F*_5, 12_ = 0.401	0.8388
MCHC (g/dL)	27.63 ± 0.43	27.20 ± 0.55	27.50 ± 0.61	27.40 ± 0.16	27.80 ± 0.26	28.23 ± 0.44	*F*_5, 12_ = 0.680	0.6469
Platelets (×10^9^/L)	729.3 ± 110.70	901.7 ± 72.30	565.0 ± 70.36	839.3 ± 85.99	812.0 ± 96.29	443.3 ± 125.30^a^	*F*_5, 12_ = 3.443	0.0368
LYM (%)	51.83 ± 6.68	67.63 ± 9.27	72.17 ± 2.53	58.50 ± 4.29	56.87 ± 4.76	10.25 ± 1.95^b^	*F*_5, 11_ = 11.13	0.0005

^*c*^ *P* < 0.0001. ^*b*^*P* < 0.001. ^*a*^*P* < 0.05 compared to CFA group (One-way ANOVA analysis followed by Dunnett multiple comparison test).

**Table 2:** Serum chemistry analysis of arthritic rats in the therapeutic study.

Parameters	IFA	CFA	PSRE 30	PSRE 100	PSRE 300	Dex	*F*	*P* Value
TOTAL PROT (g/L)	72.37 ± 0.97	72.27 ± 3.34	59.73 ± 15.53	59.93 ± 13.05	63.53 ± 10.48	52.97 ± 19.58	*F*_5, 12_ = 0.384	0.8504
ALT (U/L)	125.3 ± 17.54	121.8 ± 12.94	149.6 ± 27.77	126.7 ± 42.07	114.9 ± 23.16	103.4 ± 42.25	*F*_5, 12_ = 0.264	0.9243
AST (U/L)	350.4 ± 39.30	272.1 ± 34.76	314.3 ± 93.00	302.5 ± 89.03	315.7 ± 63.16	274.1 ± 109.6	*F*_5, 12_ = 0.146	0.9774
GGT (U/L)	6.00 ± 0.00	6.00 ± 0.00	9.50 ± 3.50	6.00 ± 0.00	6.33 ± 0.33	8.17 ± 2.17	*F*_5, 12_ = 0.778	0.5842
T-BIL (*µ*mol/L)	2.90 ± 0.00	2.90 ± 0.00	4.20 ± 1.30	2.90 ± 0.00	2.90 ± 0.00	3.33 ± 0.43	*F*_5, 12_ = 0.880	0.5232
D-BIL (*µ*mol/L)	1.60 ± 0.00	1.60 ± 0.00	2.23 ± 0.63	1.60 ± 0.00	1.60 ± 0.00	2.13 ± 0.48	*F*_5, 12_ = 0.866	0.5312
I-BIL (*µ*mol/L)	1.30 ± 0.00	1.30 ± 0.00	1.97 ± 0.67	1.30 ± 0.00	1.30 ± 0.00	1.20 ± 0.06	*F*_5, 12_ = 1.074	0.4216
CREATININE (mmol/L)	60.71 ± 0.88	39.47 ± 8.43	47.00 ± 15.85	43.70 ± 11.81	48.70 ± 11.79	48.70 ± 7.80	*F*_5, 12_ = 0.461	0.7977
UREA (mmol/L)	6.52 ± 1.35	7.50 ± 1.57	7.74 ± 2.22	8.24 ± 2.52	8.44 ± 2.57	5.43 ± 1.80	*F*_5, 11_ = 0.306	0.8998

## Data Availability

The datasets generated during and/or analyzed during the current study are available from the corresponding author on reasonable request.
